# Supplementary material for: MINTIE: identifying novel structural and splice variants in transcriptomes using RNA-seq data
Source: Genome Biol. 2021 Oct 22;22:296. doi: 10.1186/s13059-021-02507-8 (PMC8532352; doi:10.1186/s13059-021-02507-8)
Supplement: Supplementary file 1 — Additional file 1:. Supplementary Figures, Tables and Notes [file 13059_2021_2507_MOESM1_ESM.pdf]

Additional file 1 - Supplementary Information

MINTIE: identifying novel structural and splice variants in transcriptomes using RNA-seq data

Marek Cmero[1,2,3], Breon Schmidt[1,2,4], Ian J. Majewski[5,6], Paul G. Ekert[1,2,7,8], Alicia Oshlack[1,2,4]*, Nadia M. Davidson[1,2,4]*

1. Peter MacCallum Cancer Centre, Melbourne, Vic, Australia.
2. Murdoch Children's Research Institute, Parkville, Australia.
3. Sir Peter MacCallum Department of Oncology, The University of Melbourne, Parkville, Australia.
4. School of BioSciences, University of Melbourne, Parkville, Australia.
5. Walter and Eliza Hall Institute, Parkville, Australia.
6. Faculty of Medicine, Dentistry and Health Sciences, University of Melbourne, Parkville, Australia.
7. Children's Cancer Institute, UNSW, Sydney, Australia.
8. Department of Paediatrics, University of Melbourne, Parkville, Australia.

* Joint corresponding authors, supervised this project equally

Supplementary Figures

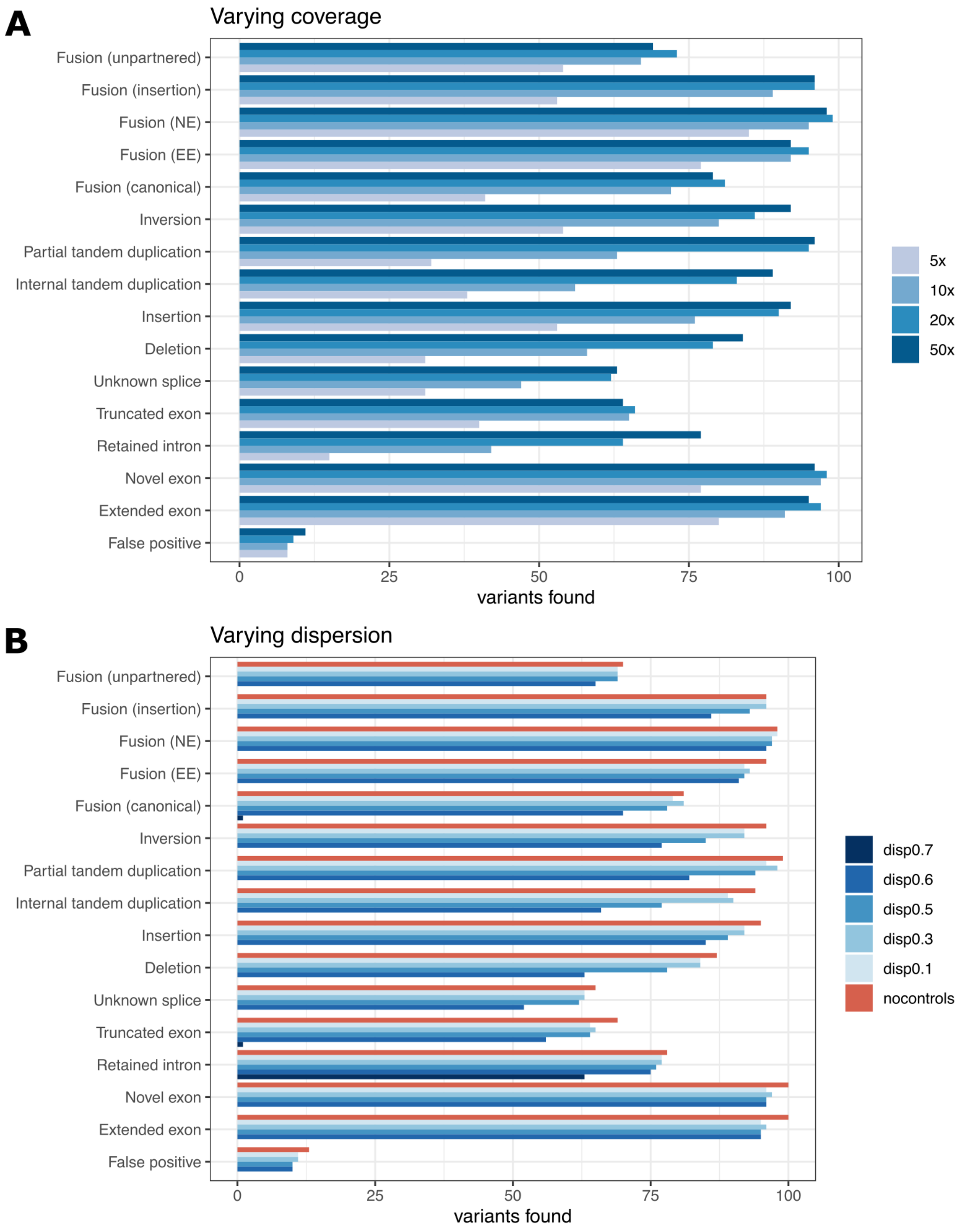

Fig. S1 | *Variants detected in simulations with varying coverage **(A)** and varying dispersion **(B)**.*

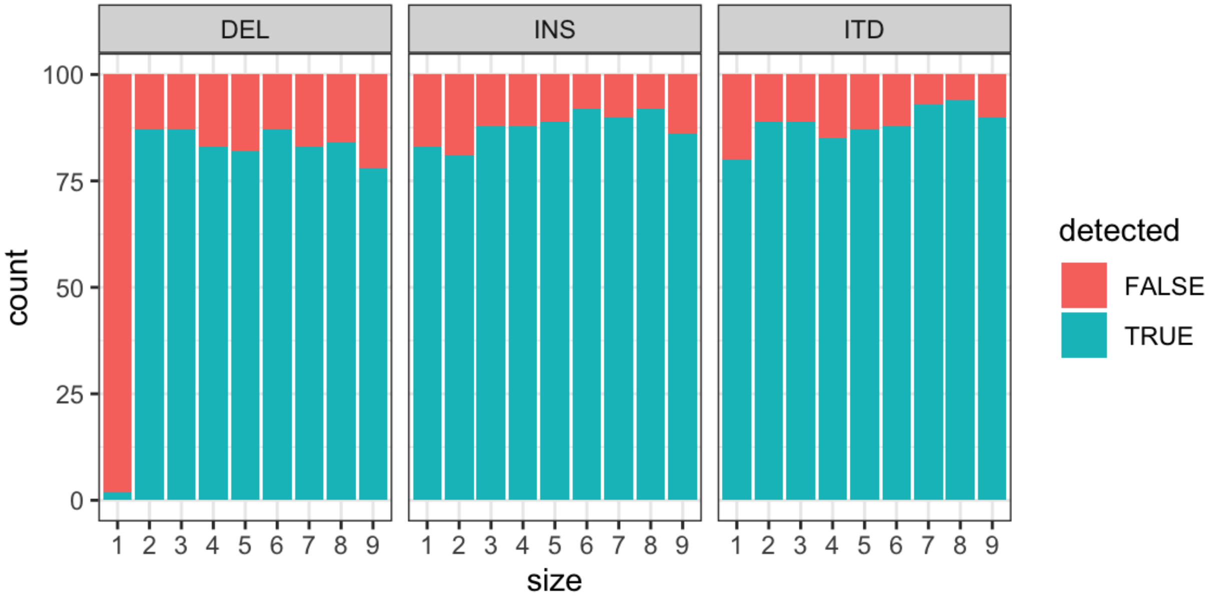

Fig. S2 | Number of deletions, insertions and ITDs detected by MINTIE at variant sizes 1-9, with 100 variants generated per variant type and size category.

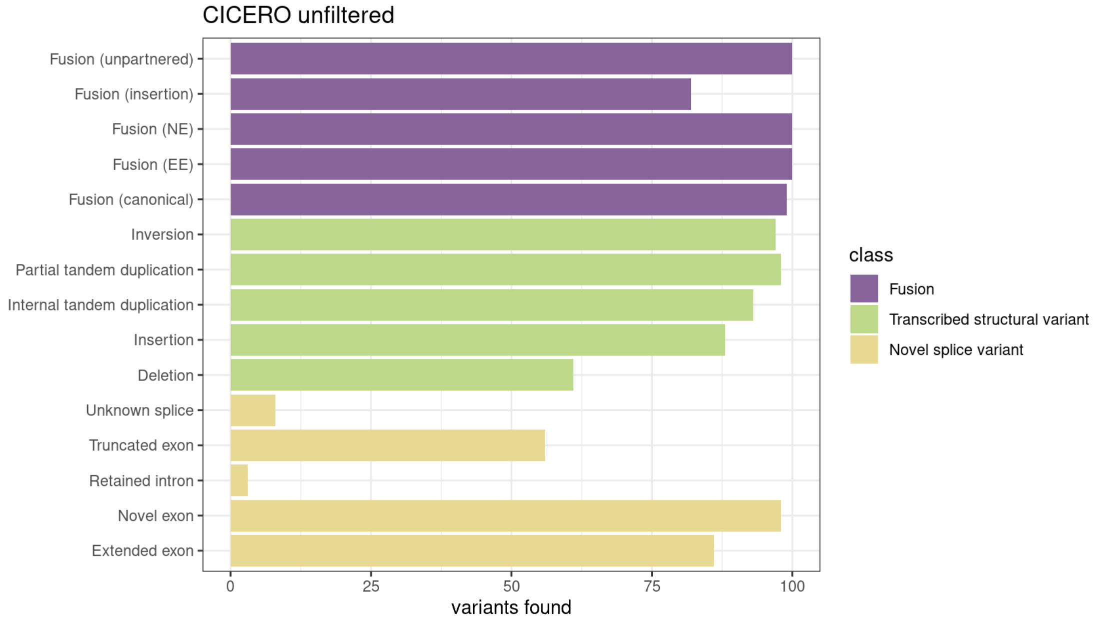

Fig. S3 | *Results of the unfiltered output from the CICERO fusion caller on the simulated data. In addition, 596 false positive calls were identified in the unfiltered output (not shown).*

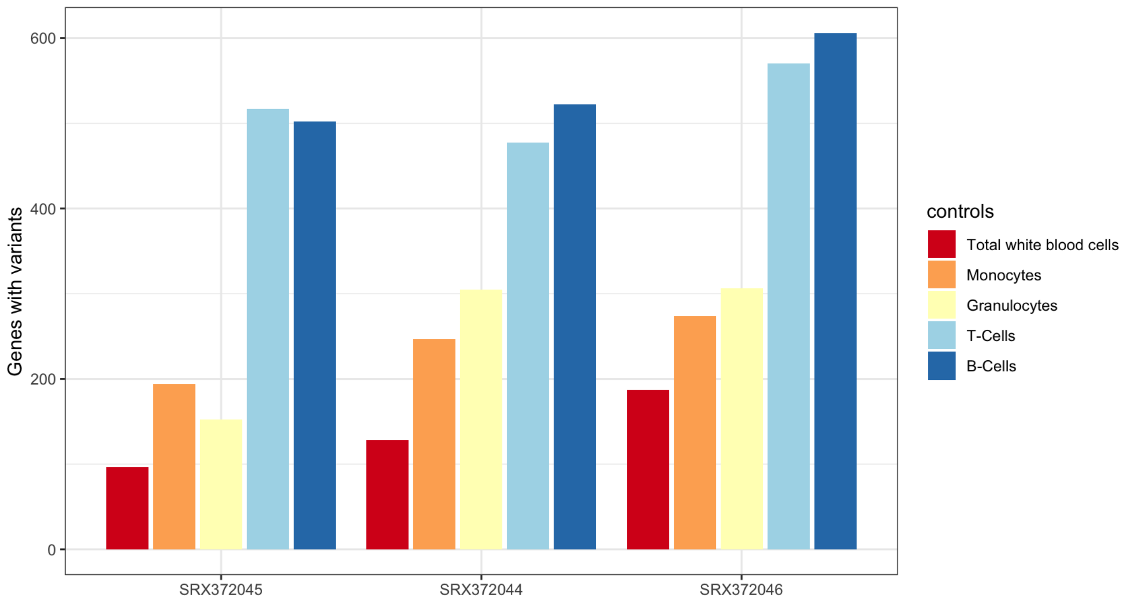

***Fig. S4* |** *Number of genes with variants found in three Leucegene total white blood cell (TWBC) samples with different normal cell types as controls. Controls are ordered by total variants found across all three samples with controls of the same type (TWBCs) resulting in the fewest variant genes. TWBCs consisted of two control samples, with all other control types consisting of five.*

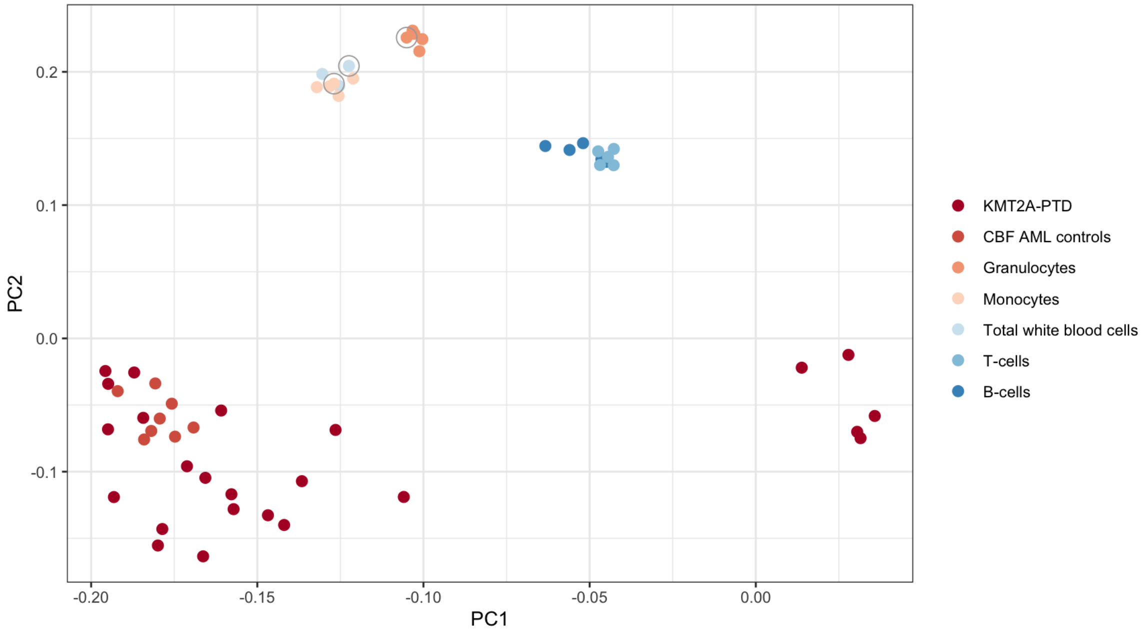

Fig.

***Fig. S5* |** *PCA plot of KMT2A-PTD cohort, compared with selected CBF AML controls, and Leucegene normals (granulocytes, monocytes, total white blood cells, T-cells and B-cells), derived from Salmon quantification of the top 500 most variable genes. The reduced control set is circled.*

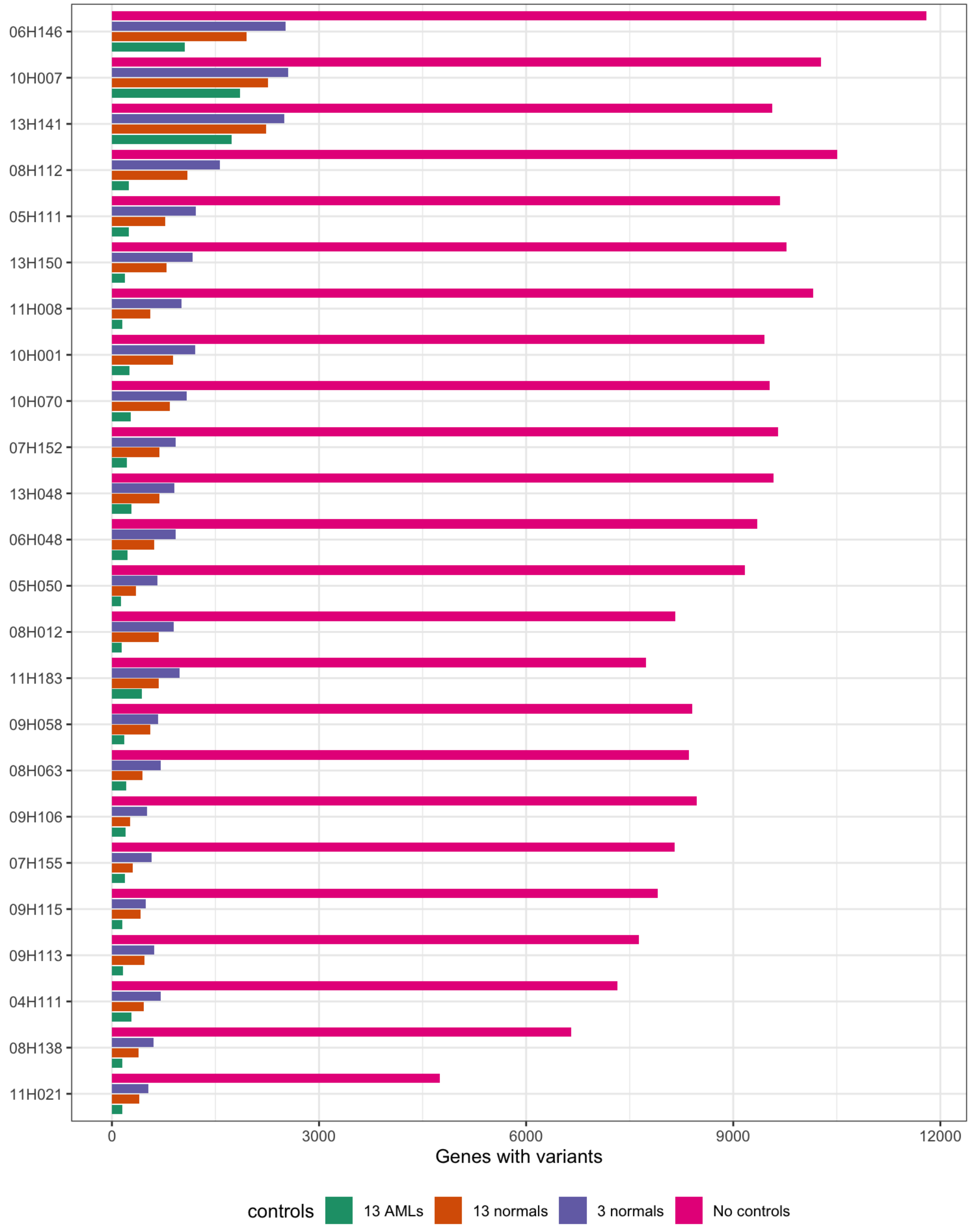

Fig. S6 | *Number of variant genes found in the KMT2A-PTD cohort (24 Leucegene samples containing KMT2A alterations) when using three different Leucegene control groups: a randomly selected set of 13 AMLs, 13 normals, 3 normals (subset of the 13) and no controls.*

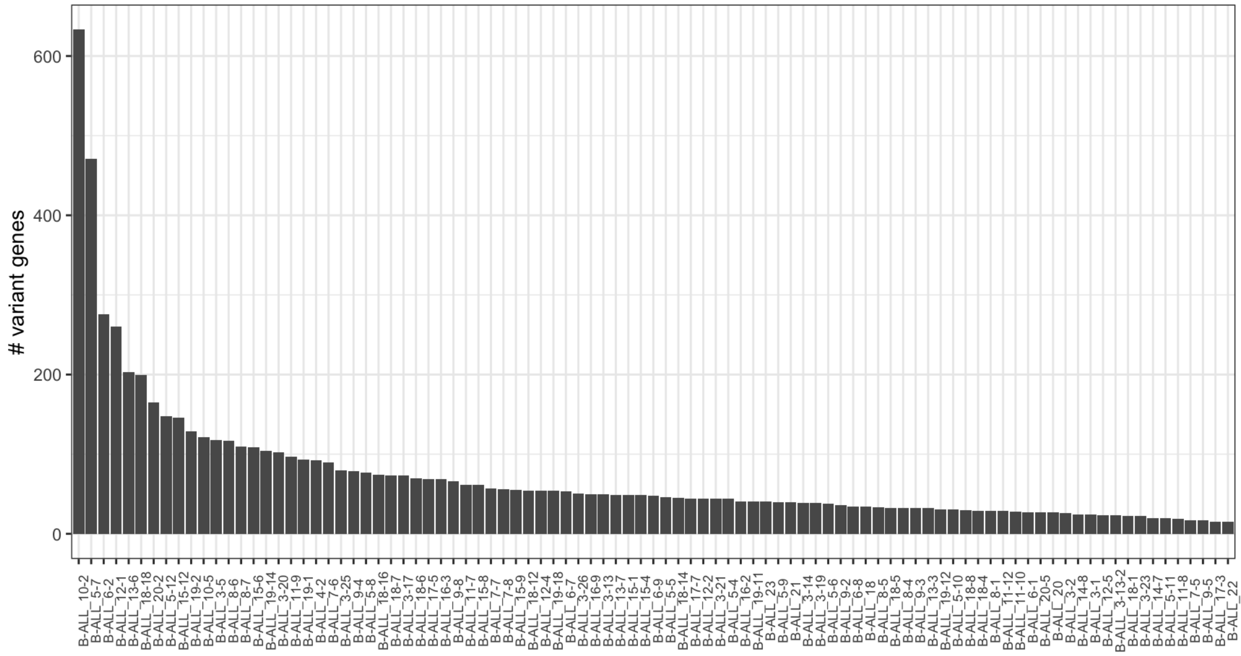

Fig. S7 **|** *Number of variant genes found per sample in RCH B-ALL cohort.*

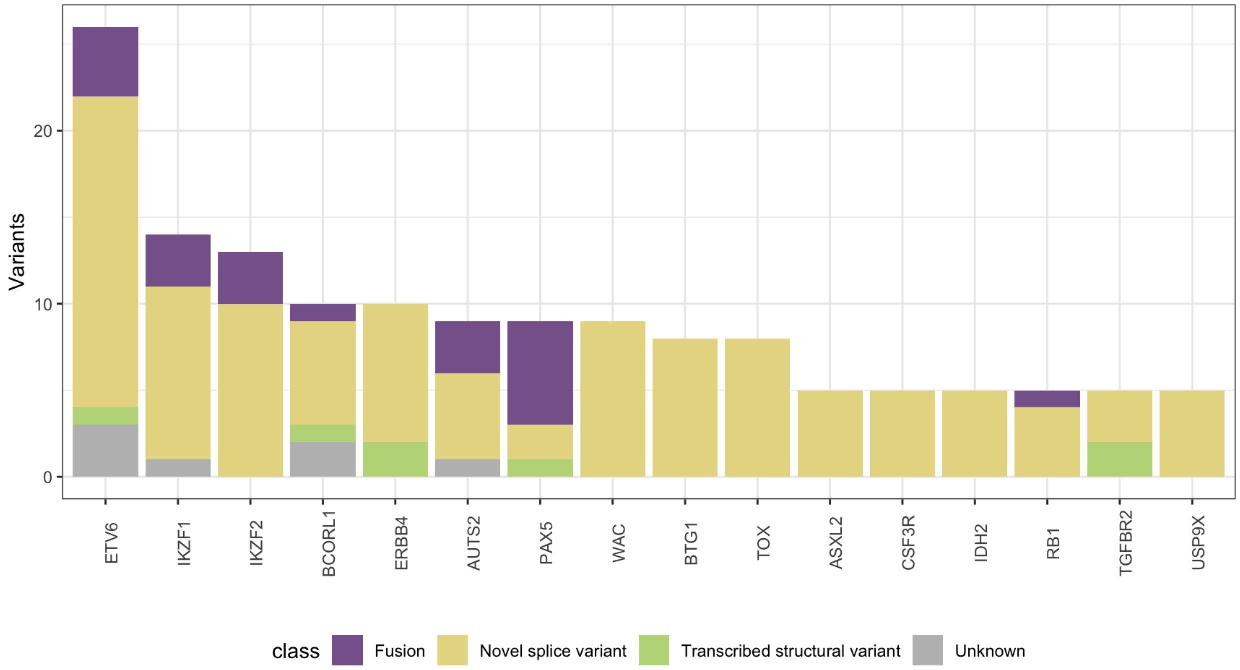

Fig. S8 **|** *The number of variants found in ALL-associated genes across the RCH B-ALL cohort.*

Supplementary Tables

Table S1 **|** *Validated KMT2A-PTDs from a prior study detected by MINTIE in a 24-sample Leucegene cohort run against 3 Leucegene normals (one each of monocytes, granulocytes and total white blood cells), 13 Leucegene normals (5 monocytes, 5 granulocytes and 3 total white blood cells), a randomly selected cohort of 13 Leucegene CBF AMLs with no known KMT2A rearrangement and no controls. Coverage obtained from Audemard et al. In cases where multiple PTDs were detected in the same patient, the highest min coverage was used.*

Patient	3 normals	13 normals	13 AMLs	No controls	Coverage
07H152	Y	Y	N	Y	158
09H115	Y	Y	N	Y	125
06H146	Y	Y	Y	Y	87
05H111	Y	Y	Y	Y	79
11H021	Y	Y	N	Y	63
05H050	Y	Y	Y	Y	58
13H150	N	Y	N	Y	58
13H048	Y	Y	Y	Y	57
10H070	Y	Y	Y	Y	53
10H007	Y	Y	Y	Y	50
09H106	Y	Y	Y	Y	49
13H141	Y	Y	Y	Y	45
09H058	Y	Y	Y	Y	29
07H155	Y	Y	N	Y	23
08H112	Y	Y	Y	Y	22
09H113	Y	Y	Y	Y	17
11H183	N	N	N	N	16
08H012	N	N	N	N	15
08H138	N	N	N	Y	15
11H008	N	N	N	Y	13
06H048	N	N	N	Y	10
08H063	N	N	N	N	6
10H001	N	N	N	Y	6
04H111	N	N	N	N	3
**Found (of 24)**	**11**	**16**	**15**	**19**	

Table S2 | *Novel variants found in the B-ALL cohort in clinically relevant genes. All locations are hg38. RB1 and ETV6 variants were both detected in 3 samples total.*

**Gene**	**Variant type**	**Samples affected**	**Novel splice junction**	**Sequence queried by SeqOthello**	**TCGA hit**
RB1	Unpartnered fusion	3	chr13:48381444-48644164	TAGAACGATGTGAACATCGAATCATGGAATCCCTTGCATGGCTCTCAAGTCAGTTCCTGCCCCACTGCCCCACAGAAGTGTTTTCTGATGTGCT	
RB1	Unpartnered fusion	1	chr13:48381444-48647695	TAGAACGATGTGAACATCGAATCATGGAATCCCTTGCATGGCTCTCAAGCCTTGTATACACTCAATATGCAAGAAGCCCTGGAAGTTCCCAAGGT	
RB1	Unpartnered fusion	1	chr13:48381444-48530084	TAGAACGATGTGAACATCGAATCATGGAATCCCTTGCATGGCTCTCAAGACAGAGTTTTGCCATGTTGTCCCGGCTGGTCTCTAACTCCTGGGCT	1 BRCA, 1 ESAD
IKZF1	PTD	1	chr7:50382708-50382540	CTGCCGCCGGAGGGACGCCCTCACTGGCCACCTGAGGACGCACTCCGGAGAACGGCCCTTCCAGTGCAATCAGTGCGGGGCCTCATTCACCCAGA	
PAX5	PTD	1	chr9:37020805-37002647	ATCACGTCCCCCAGCGCCGACACCAACAAGCGCAAGAGAGACGAAGGACATGGAGGAGTGAATCAGCTTGGGGGGGTTTTTGTGAATGGACGGCC	
ETV6	Skipped exons	1	chr12:11752580-11869424	AGCGCTCAGGATGGAGGAAGACTCGATCCGCCTGCCTGCGCACCTGCATAACTGTGTCCAGAGGACCCCCAGGCCATCCGTGGATAATGTGCACC	1 LSCC
ETV6	Skipped exons	2	chr12:11752580-11890941	AGCGCTCAGGATGGAGGAAGACTCGATCCGCCTGCCTGCGCACCTGCGTTTATGAAAACCCCAGATGAAATCATGAGTGGCCGAACAGACCGTCT	
ETV6	Skipped exons + novel exons	1	chr12:11869970-11931173	TGTCTCCCCGCCTGAAGAGCACGCCATGCCCATTGGGAGAATAGCAGGTCCCATCCCATCCGAGTCTCAACAGAAACATCACCTCCCCAGGGAGA	

Table S3 | *Novel candidate variants found in the rare disease data.*

Patient	Genetic Diagnosis	Gene	Location 1	Location 2	Size	Variant Type	rs ID
C5	Strong candidate gene	DMD	chrX:32287529	chr8:65268274, chr8:65267608, chr8:65194163	N/A	Unpartnered Fusion	
N24	No strong candidates	DMD	chrX:31121428	chrX:31121443	17	3' UTR Deletion	rs763028610
N9	No strong candidates	KLHL9	chr9:21335327	chr9:21335339	12	5' UTR Deletion	rs201092918
N23	No strong candidates	KLHL9	chr9:21335327	chr9:21335339	12	5' UTR Deletion	rs201092918
D9	Diagnosed	LDB3	chr10:86735779	chr10:86735786	7	3' UTR Deletion	rs746342719
N24	No strong candidates	LDB3	chr10:86735779	chr10:86735786	7	3' UTR Deletion	rs746342719
N11	No strong candidates	LDB3	chr10:86735779	chr10:86735786	7	3' UTR Deletion	rs746342719
D13	Diagnosed	LDB3	chr10:86735779	chr10:86735786	7	3' UTR Deletion	rs746342719
N6	No strong candidates	VAPB	chr20:58445685	chr20:58445701	16	3' UTR Deletion	rs138225455

Supplementary Notes

***Note S1* |** *List of Leucegene samples analysed.*

Core binding factor cohort: 03H065, 03H083, 03H095*, 03H109, 03H112, 04H030*, 04H061*, 04H091*, 05H042*, 05H099*, 05H113, 05H118*, 05H136*, 05H184*, 06H020, 06H035*, 06H115*, 07H099*, 07H137*, 07H144, 08H034, 08H042, 08H072, 08H081, 08H099, 09H016, 09H040, 09H066, 10H008, 10H030, 10H119, 11H022, 11H104, 11H107, 11H179, 12H042, 12H044, 12H045, 12H098, 12H165, 12H166, 12H180, 12H183, 13H066, 13H120, 13H169 (*used as controls).

NUP98-NSD1 cohort: 03H041, 05H034, 05H163, 08H049, 10H038, 11H027, 11H160.

KMT2A-PTD cohort: 05H050, 09H113, 09H115, 11H021, 08H012, 08H112, 11H008, 05H111, 06H146, 04H111, 06H048, 07H152, 07H155, 08H063, 08H138, 09H058, 09H106, 10H001, 10H007, 10H070, 11H183, 13H048, 13H141, 13H150

***Note S2* |** *Samples used as controls in the RCH B-ALL analysis.*

B-ALL3-1, B-ALL3-2, B-ALL5-8, B-ALL5-10, B-ALL5-11, B-ALL7-7, B-ALL8-1, B-ALL9-2, B-ALL9-5, B-ALL11-8, B-ALL12-4, B-ALL14-7, B-ALL14-8, B-ALL16-2, B-ALL16-3, B-ALL17-3, B-ALL18-14, B-ALL9-8, B-ALL10-2, B-ALL6-9, B-ALL11-10, B-ALL15-9, B-ALL19-11, B-ALL6-1, B-ALL18-4, B-ALL13-7, B-ALL19-12, B-ALL6-8, B-ALL3-26, B-ALL-22, B-ALL16-9, B-ALL3-20, B-ALL3-25, B-ALL18-7
