# Supplementary material for: MINTIE: identifying novel structural and splice variants in transcriptomes using RNA-seq data
Source: Genome Biol. 2021 Oct 22;22:296. doi: 10.1186/s13059-021-02507-8 (PMC8532352; doi:10.1186/s13059-021-02507-8)
Supplement: Supplementary file 2 — Additional file 2:. Review history [file 13059_2021_2507_MOESM2_ESM.docx]

**Review history**

**First round of review**

**Reviewer 1**

The manuscript " MINTIE: identifying novel structural and splice variants in transcriptomes using RNA- seq data" by Cmero et al. describes a method for identifying transcribed structural variants (TSVs) in genomes based on RNA-seq leveraging de novo assembly.  Unlike most similar methods that tend to be focused on finding limited classes of TSVs, MINTIE intends to be a catch-all method, with the goal of identifying all case sample relevant variants that are significantly differentially abundant in the case sample as compared to a set of control samples.  The authors benchmark MINTIE on simulated data in comparison to 8 other methods and demonstrate MINTIE to be accurate in identifying variants across all classes, in stark contrast to the other methods. Then, application of MINTIE is demonstrated on a multitude of real disease and normal transcriptome samples and shown capable of discovering previously unreported disease-related variants.

This work was very well done and the manuscript reads beautifully. The method appears to be quite flexible and not tied to any specific de novo assembly method, and the authors do an excellent job of highlighting the limitations of the method.  There are a few aspects of the work that would benefit from further clarification and additional insights.  I describe these issues below.

Regardless of whether the method is truly the best approach to tackling the aspect of diverse TSV identification, it clearly works and has demonstrated utility on multiple disease-related data sets, and I truly welcome the developments herein.  I expect this work and the software to be well received by the community and it's a clear demonstration of the value of de novo transcript assembly for exploring transcript structural variations relevant to studies in cancer and broader diseases with genetic underpinnings.


Major:

The authors mention DE-kupl as one of the methods that operates in this realm of TSV detection.  It's aims are highly similar to those of MINTIE with one of the main differences being that it operates based on kmer counts rather than de novo assemblies.  It wasn't used as one of the comparators solely because it requires multiple case samples and multiple control samples, whereas Mintie and the other methods can leverage only a single case sample.  This primary restriction is likely due to the requirements of DE-kupl for differential expression analysis in needing multiple case samples and control samples.  However, I expect this difference could be easily overcome by using an identical or nearly identical invocation of edgeR as performed by MINTIE.  I don't expect the authors here to hack DE-kupl into being able to leverage this modality, but it does raise some important questions regarding the value of the MINTIE de novo assembly approach as compared to this kmer-based strategy.   It would be helpful for the authors to elaborate on expectations here in the manuscript, and to highlight those variants they expect to be able to capture via de novo assembly that would not be suitably captured using such a kmer based approach as implemented in DE-kupl.  Note, if there is a straightforward way to include DE-kupl in the benchmarking effort (perhaps discuss with DE-kupl developers/support), then that would add great value to the work here.

The only benchmarking that was performed involved simulated data where all variant classes were modeled at high (50x) coverage.  While this simulation does demonstrate MINTIE's capabilities for finding diverse TSVs in comparison to the alternative methods, it doesn't appear to reflect the dynamic range of expression values that might be expected for accurate application to real samples.  It clearly indicates that MINTIE has good sensitivity for diverse variants when they present at reasonably high levels of coverage, but I would expect that sensitivity to rapidly deteriorate as coverage decreases due to the needs of de novo transcript assembly.  If MINTIE retained high sensitivity (and overall accuracy) for diverse variant classes at both low and moderate to high coverage, then that would better justify the comment in the discussion section that "we propose that MINTIE could be used as a single tool to detect any large alterations, replacing the need to run multiple tools".

Benchmarking of MINTIE in comparison to the alternative methods is restricted to the simulated data.  However, MINTIE is further benchmarked on its own using real data with known TSVs under section " MINTIE identifies known fusions and ITDs in Leucegene samples". It would benefit the manuscript to also benchmark the competing methods on these data as well so as to better understand the merits of MINTIE as compared to the alternatives on these real (as opposed to simulated) benchmarking data.

A critical component of MINTIE's variant detection algorithm relies on the ability to detect variant transcript regions as differentially expressed.  There are three primary variables that I expect should impact this: sequencing depth, length of the variant feature, and number of control samples.  MINTIE is stated as being limited to detecting variants that are at least 7 bases in length, but this is operationally defined by a program parameter rather than as a known inherent characteristic of the method. It would be useful to know the actual empirical detection limits of MINTIE.  This could be readily explored via the authors simulation toolkit, presumably.  To address how the number of control samples impacts the number of detected variants, the authors could more comprehensively explore these effects under the section on background variants - such as randomly choosing sets of 1..N controls and exploring the number of and variability in the number of variants detected under the corresponding number of controls.  These could be limited to the 'best' controls, avoiding those that are demonstrated to lead to increases in variant counts due to sample divergence - so using 'best case scenario' controls.  Related to this, and a relatively minor point, is the issue that execution of MINTIE on simulated data lacks biological replicates, and so the differential expression analysis had to be run using a fixed dispersion parameter (0.1).  Running any differential expression analysis with edgeR and having to use a fixed dispersion parameter is something that is greatly frowned on in the community, but it's necessary in the case you don't have 'biological replicates' for which to computationally estimate dispersion. Again, this is likely the reason for why DE-kupl doesn't allow execution without biological replicates.  Including some supplementary information on how setting this dispersion parameter impacts MINTIE's detection accuracy on the simulated data would be useful to include, since in application, variability in expression among control samples contributes to feature identification.

It's indicated in the manuscript that multiple case samples can be used in addition to multiple controls and that they can be run in parallel.  This suggests to me that the multiple cases would be run individually and separately, each in comparison to the N number of controls, rather than treating the multiple case samples as 'biological replicates' in a single analysis against the N controls (as would be performed by DE-kupl in the comparable workflow).  Can the authors elaborate on this aspect and provide best recommendations?  If such as multi-case sample situation is available, presumably it would enable for more direct comparisons to the DE-kupl method.


Minor:

It would help for the authors to elaborate more on how they were able to infer the various TSV categories based on the results from these various programs.  There was clearly a lot of work involved to do this, as most of these related software tools do not output lists of TSVs with categories assigned.  While the methods do expand on how this was done, it would help to add a few sentences in the main text to emphasize that there is a lot of work here in downstream interpretation and classification into these different TSV event categories so it'll be better appreciated.  Also, while I haven't had time to run MINTIE myself, it could be stated early on whether or not it provides such categorical lists of TSVs in its output or whether interpretation and analysis is required by the user.

p27 - adjust the wording to indicate that the Rapid RNA-seq pipeline is the St. Jude's pipeline and not something different.

p11, line 29:  ECCs used instead of ECs as elsewhere

**Reviewer 2**

The manuscript entitled "MINTIE: identifying novel structural and splice variants in transcriptomes using RNA-seq data" describes an analytical pipeline which detect structural and splice variants in transcriptomes by a de novo assembly using SOAPdenovo-Trans of a case sample, merging assembled transcripts with the references assembled by CHESS v2.2, quantifying expression for both case and control samples using Salmon, and retain non-reference (termed "novel" by the authors) transcripts that are over-expressed only in case, and finally mapping the candidate transcripts to the genome and performing annotation. The accuracy of MINTIE was based on an analysis of simulated data which  shows MINTIE can predict diverse classes of non-reference transcripts with accuracy comparable to those designed specifically for gene fusion or alternative splicing. Additionally, the authors presented MINTIE's recall rate of known fusions/FLT3 ITD in 77 AMLs and showcased novel fusions in 87 pediatric B-ALLs as well as DMD fusion found in a rare disease sample. Overall, incorporating "control" samples which enables assessing differential expression in case sample versus "control" is the most unique design of MINTIE. However, the impact of this method, i.e.  is uncertain. Specific comments are listed below.

Major issues:
a)     The most desirable feature of MINTIE is its ability to capture aberrant splicing and gene fusions in a single analysis instead of running methods specifically designed for gene fusion and alternative splicing. The superiority of MINTIE is demonstrated via its performance on simulated data of 15 different classes of variants presented in Figure 3.  However, this result only shows that MANTIE has great performance on this simulated data and the design of the simulation, which gives high-coverage (50X coverage in a heterozygous fashion, total coverage of 100X, line 28-34, page 7) of the variant, do not match the expression profile of many pathogenic fusions or transcripts as these variants may not reach the 50X threshold in simulated RNA-seq data. Therefore, the statistics presented in Figure 3 may not reflect its performance on real cancer specimen. In fact, the false negative rate is somewhat reflected upon the less-than optimal performance of the 77 AMLs presented in the section "MINTIE identifies known fusions and ITDs in Leucegene samples" as the missed CBFB-MYH11(2 out of 26) and KMT2A-PTD (8 out 24) were due to the lower expression level based on the authors evaluation. Comparison of MINTIE with other fusion/isoform detection methods on real disease samples is needed to convince the user that using MINTIE alone can achieve the same sensitivity as running a combo of fusion + isoform detection.
b)     The authors need to present MINTIE's performance on solid and brain tumors, many of which have much lower tumor purity than leukemia samples presented in the manuscript. This will allow a more comprehensive analysis regarding MINTIE's performance on tumor samples.
c)     SOAPdenovo assembly, or any other de novo assembly algorithms, may encounter issues when the fusion breakpoint occurs at the highly repetitive regions. For example, our test run of MANTIE using the Nalm6 cell line RNA-seq data (accessible as a demo sample for CICERO at https://www.stjuderesearch.org/site/lab/zhang/cicero) failed to detect the IGH-DUX4 fusion. In this test, we used 13 control RNA-seq (normal samples: 5 granulocytes, 5 monocytes, 3 total white blood cells) documented in MANTIE which produced a total of 1,870 variants. Limitation of the assembly algorithm needs to be noted so that the users are aware about the potential false negatives.
d)     In the section "MINTIE detects a low number of background variants", the authors presented the variants detected a set of 23 non-cancer samples (Figure 4). However, there is a 10-fold differences within the samples and it is unclear what are the factors that contribute to the false positive rate. It should also be noted that nailing down the candidate fusions in the B-ALL samples presented in the section of "MINTIE identifies novel transcriptomic variants in B-ALL" has a dependency on knowledge-based ancillary data. First, control samples were from the same batch of the RNA-seq with a prior knowledge of RNA fusion status; second the candidate variants require filtering of candidate genes identified from prior pan-cancer studies. These constraints will greatly limit the applicability of MINTIE.
e)     Novelty of the B-ALL findings presented in XXX is over-stated The unpartnered RB1 recurrent fusion was detected in the pan-TARGET study (Ma et al, Nature 2018) as loss-of-function structural variations (either by translocation or deletion) of RB1 in both T-lineage and B-lineage ALL. The variant can be easily explored on the recently published GenomePaint portal (https://genomepaint.stjude.cloud/, Zhou et al, Cancer Cell 2021). Similarly PAX5 PTD was first reported in 2007 (Mullighan et al, Nature 2007, Figure 2), and intra-genic deletion in ETV6 causing alternative splicing reported in the manuscript can be found in 2011 (Zhang et al, Blood 2011, Figure 4). Therefore, these findings are not novel.


Minor issues
a)     Figure 5. The y-axis label is difficult to read. The number of supporting reads for each splice junction is difficult to read. Panel B has two arcs representing the same splice junction but with varying read count. It will be good to document why there are different read counts in Figure legend
b)     References on the second paragraph of Results (page 5-6, starting with line 46) appear to be incorrect. For example: SOAPdenovo-Trans was first referenced on line 48, page 5. However, the reference (#28) it cited was referring to a different program DE-kupl. DE-kupl was cited using the old bioarchive paper instead of the Genome Biology publication. The references for CHESS and Salmon appear to be incorrect as well.
c)     In the paragraph on page 14, the authors noted "The top recurrent genes were ETV6 (26 variants across 8 samples), IKZF2 (11 variants across 2 samples) and PAX5 (9 variants across 6 samples) (Supplementary Figure 5). It should be noted that the number of variants per sample is >1 in all three genes and in particular there are a total of 11 variants in 2 samples. The authors need to explain the genetic cause of these variants. In particular, IKZF2 was previously noted to have intra-genic deletion in hypodiploid ALL (Holmfeldt et al, Nature Genetics 2013). The high number of SVs presented in Supplementary Figure 5 is a surprise and the authors need to explain the potential genetic events leading to the high number of SVs in this gene in the two samples.
d)     Memory requirement may exceed 90-100GB based on our test using the RNA-seq data from CICERO benchmark data set which requires 151-283 GB memory .We also noted false negative on MINTE in detecting lowly expressed gene fusions/ITD.

**Authors’ response to reviewers**

We would like to thank the reviewers for their insights and suggestions. We have made several improvements to the pipeline, including support for running without controls, classification of intragenic rearrangements, leniency for motif checking (allowing splice boundary motif mismatches due to SNPs/SNVs), variant sequence output (40bp sequence(s) spanning each variant boundary) as well as numerous fixes and usability improvements. All samples from the original paper have been rerun through the new version of the pipeline (v0.3.5; updated from v0.2.0) to show the most up-to-date results.

Furthermore, we have addressed the major revisions with the following:

- We downsampled the simulation variant read files to explore the effect of coverage on MINTIE’s performance.
- We performed experiments with different dispersion values for the simulations.
- We explored the limits of variant size detection with new simulations.
- We have explored MINTIE’s background variant rate when varying the number of controls in the Leucegene normals cohort.
- We have run competing methods in the Leucegene validation data, and compared these to MINTIE’s results.
- We applied MINTIE to two TCGA samples previously identified as having the RB1 unpartnered fusion from SeqOthello.

Reviewer reports:

Reviewer #1: The manuscript " MINTIE: identifying novel structural and splice variants in transcriptomes using RNA- seq data" by Cmero et al. describes a method for identifying transcribed structural variants (TSVs) in genomes based on RNA-seq leveraging de novo assembly. Unlike most similar methods that tend to be focused on finding limited classes of TSVs, MINTIE intends to be a catch-all method, with the goal of identifying all case sample relevant variants that are significantly differentially abundant in the case sample as compared to a set of control samples. The authors benchmark MINTIE on simulated data in comparison to 8 other methods and demonstrate MINTIE to be accurate in identifying variants across all classes, in stark contrast to the other methods. Then, application of MINTIE is demonstrated on a multitude of real disease and normal transcriptome samples and shown capable of discovering previously unreported disease-related variants.

This work was very well done and the manuscript reads beautifully. The method appears to be quite flexible and not tied to any specific de novo assembly method, and the authors do an excellent job of highlighting the limitations of the method. There are a few aspects of the work that would benefit from further clarification and additional insights. I describe these issues below.

Regardless of whether the method is truly the best approach to tackling the aspect of diverse TSV identification, it clearly works and has demonstrated utility on multiple disease-related data sets, and I truly welcome the developments herein. I expect this work and the software to be well received by the community and it's a clear demonstration of the value of de novo transcript assembly for exploring transcript structural variations relevant to studies in cancer and broader diseases with genetic underpinnings.

Major:

1. The authors mention DE-kupl as one of the methods that operates in this realm of TSV detection. It's aims are highly similar to those of MINTIE with one of the main differences being that it operates based on kmer counts rather than de novo assemblies. It wasn't used as one of the comparators solely because it requires multiple case samples and multiple control samples, whereas Mintie and the other methods can leverage only a single case sample. This primary restriction is likely due to the requirements of DE-kupl for differential expression analysis in needing multiple case samples and control samples. However, I expect this difference could be easily overcome by using an identical or nearly identical invocation of edgeR as performed by MINTIE. I don't expect the authors here to hack DE-kupl into being able to leverage this modality, but it does raise some important questions regarding the value of the MINTIE de novo assembly approach as compared to this kmer-based strategy. It would be helpful for the authors to elaborate on expectations here in the manuscript, and to highlight those variants they expect to be able to capture via de novo assembly that would not be suitably captured using such a kmer based approach as implemented in DE-kupl. Note, if there is a straightforward way to include DE-kupl in the benchmarking effort (perhaps discuss with DE-kupl developers/support), then that would add great value to the work here.

Up to the stage of identifying differentially expressed contigs, the two methods are conceptually similar. MINTIE assembles first, then performs DE on the contigs using EC counts, while DE-kupl performs DE on kmers, and then extends the resulting kmers. However, this is only half of the MINTIE method--the other half performs annotation and filtering of variant contigs, which is not performed by DE-kupl. MINTIE’s utility is also reflected in the variant annotation and prioritisation that the pipeline output provides. Notably, DE-kupl outputs a large number of variant kmers (>130k in the EMT experiment presented in the DE-kupl paper). MINTIE (with controls), typically outputs fewer variants by about two orders of magnitude.

As the reviewer notes, given a modification to run one versus one/many analyses, DE-kupl may be able to find the same types of variants as MINTIE. To investigate this, we made minor modifications to DE-kupl’s differential expression to allow one versus one comparison, similar to how this is handled by MINTIE. As DE-kupl outputs significant extended kmers, we used a similar strategy to check performance on simulation results as with KisSplice’s output, taking all extended kmer sequences and aligning them with GMAP, then checking alignment to variant genes. These results showed that DE-kupl called every simulated variant’s gene(s) as significant, as well as 1,146 false positive genes that did not match the simulations. On closer inspection, we noted that many of the assembled kmers were exact matches to the reference. This may have been due to minor differences in the references used in the simulation and the reference masking used within DE-kupl.

To benchmark DE-kupl’s variant calls, we took the extended kmers from DE-kupl and ran them through MINTIE’s annotation and refinement scripts, and found that even hits in the correct gene did not have the correct variant classification (see figure below, structured in the same way as in the new Figure 3B). On manual inspection, many of these contigs aligned poorly to the genome. As these results involved using an altered version of DE-kupl that it was not calibrated for, we felt it was not fair to benchmark the tool on par with MINTIE and other approaches, and therefore opted not to show these results in the manuscript.

One interesting aspect of performing these experiments, is that it shows that one could actually use DE-kupl as an ‘assembler’ for MINTIE’s input. The resulting extended kmers from DE-kupl can be used as assembly input to the MINTIE pipeline and subsequent annotation and filtering could be performed. The tools could thus be used in complement. This may serve as a viable strategy for variant filtering and discovery.

To address the reviewers comment in the manuscript we have noted conceptual similarities with DE-kupl in the Introduction. We also now mention why DE-kupl was not run in the section “MINTIE detects more types of variants than other tools” in Results.

2. The only benchmarking that was performed involved simulated data where all variant classes were modeled at high (50x) coverage. While this simulation does demonstrate MINTIE's capabilities for finding diverse TSVs in comparison to the alternative methods, it doesn't appear to reflect the dynamic range of expression values that might be

expected for accurate application to real samples. It clearly indicates that MINTIE has good sensitivity for diverse variants when they present at reasonably high levels of coverage, but I would expect that sensitivity to rapidly deteriorate as coverage decreases due to the needs of de novo transcript assembly. If MINTIE retained high sensitivity (and overall accuracy) for diverse variant classes at both low and moderate to high coverage, then that would better justify the comment in the discussion section that "we propose that MINTIE could be used as a single tool to detect any large alterations, replacing the need to run multiple tools".

To study the effect of coverage on MINTIE’s variant detection ability, we downsampled the simulation data at four variant-coverage levels: 20x, 10x and 5x. Supplementary Figure 1A shows the performance at these coverage levels. At 50x (the original simulation coverage), MINTIE detected 1,293 out of 1500 variants, this dropped minimally to 1,273 at 20x, with a modest drop at 10x (1,098) and further drop at 5x (769). These results indicate that MINTIE performs well at low coverage, and even detects most variants at low coverage. We now refer to these results in the section: “MINTIE detects more types of variants than other tools”.

We have also removed the comment, “we propose that MINTIE could be used as a single tool to detect any large alterations, replacing the need to run multiple tools” in the Discussion section, clarifying that MINTIE’s innovation is being a catch-all tool capable of rare and unusual variant discovery.

3. Benchmarking of MINTIE in comparison to the alternative methods is restricted to the simulated data. However, MINTIE is further benchmarked on its own using real data with known TSVs under section " MINTIE identifies known fusions and ITDs in Leucegene samples". It would benefit the manuscript to also benchmark the competing methods on these data as well so as to better understand the merits of MINTIE as compared to the alternatives on these real (as opposed to simulated) benchmarking data.

We have selected three competing methods to compare with for the Leucegene validation cohort: Arriba (a representative fusion caller), SQUID (a representative large TSV caller) and TAP (a general purpose caller). We present the results in a new figure (Figure 4A). While this figure shows that MINTIE is no more sensitive than state-of-the-art tools for detecting known, well defined variants, the strength of the approach is in detecting other variant types that, by definition, do not have a number of well-known and validated truth sets available to benchmark against. The Leucegene validation cohort incorporates only three of the 15 variant types that can be detected by MINTIE, and thus represents only a fraction of MINTIE’s utility.

4. A critical component of MINTIE's variant detection algorithm relies on the ability to detect variant transcript regions as differentially expressed. There are three primary variables that I expect should impact this: sequencing depth, length of the variant feature, and number of control samples. MINTIE is stated as being limited to detecting variants that are at least 7 bases in length, but this is operationally defined by a program parameter rather than as a known inherent characteristic of the method. It would be useful to know the actual empirical detection limits of MINTIE. This could be readily explored via the authors simulation toolkit, presumably.

To address how the number of control samples impacts the number of detected variants, the authors could more comprehensively explore these effects under the section on background variants - such as randomly choosing sets of 1..N controls and exploring the number of and variability in the number of variants detected under the corresponding number of controls. These could be limited to the 'best' controls, avoiding those that are demonstrated to lead to increases in variant counts due to sample divergence - so using 'best case scenario' controls. Related to this, and a relatively minor point, is the issue that execution of MINTIE on simulated data lacks biological replicates, and so the differential expression analysis had to be run using a fixed dispersion parameter (0.1). Running any differential expression analysis with edgeR and having to use a fixed dispersion parameter is something that is greatly frowned on in the community, but it's necessary in the case you don't have 'biological replicates' for which to computationally estimate dispersion. Again, this is likely the reason for why DE-kupl doesn't allow execution without biological replicates. Including some supplementary information on how setting this dispersion parameter impacts MINTIE's detection accuracy on the simulated data would be useful to include, since in application, variability in expression among control samples contributes to feature identification.

Regarding the effects of coverage, please see the answer to point 2 above, describing MINTIE’s performance on downsampled simulations. We also reran the simulations at four additional fixed dispersion values, as well as without controls, shown in Supplementary Figure 1B. The results indicate that increasing dispersion values has a minimal effect on the results, up until a point of very high dispersion (between 0.6 and 0.7), where a thresholding effect appears to reduce the results greatly. This indicates that data with moderate dispersion values should not affect the results significantly, as long as it is at 0.6 or below.

We have addressed the question of detection limits by performing an additional set of simulations affected by the gap size parameter (insertions, ITDs and deletions), shown in Supplementary Figure 2. We generated an additional 2,700 simulated variants, 100 per size and variant category from 1-9 bp. The results indicate that MINTIE’s detection rate is largely unaffected by variant size (apart from a deletion size of 1) and we now make a note of this in the text of the manuscript.

To address MINTIE’s performance when using different numbers of controls, we took the five leucegene granulocyte normals and ran all combinations of 1-4 controls for each case sample. These results are shown in Figure 4C. The pattern of more controls equals fewer variants for all samples and indicates fewer false positives are expected given more controls. Secondly, while four samples varied minimally, one sample displayed large variance in the number of variant genes called. On closer inspection, when compared with expression similarity in the PCA plot, highly similar control samples yielded fewer variants. As this sample had some highly similar samples, and more dissimilar samples, the different combinations of controls yielded more variable numbers of variants called. We now make a note of this in the Results section of the manuscript, titled “MINTIE is robust to varying coverage and dispersion”.

We have also added functionality to run MINTIE without controls. When this is enabled, DE is not performed (however, novel ECs are still identified from reference transcript overlap). This mode is intended to be used in cases where no appropriate controls exist, or to maximise sensitivity (at the expense of false positives). We have shown the effect that running without controls has in the KMT2A PTD cohort (Supplementary Figure 6).

5. It's indicated in the manuscript that multiple case samples can be used in addition to multiple controls and that they can be run in parallel. This suggests to me that the multiple cases would be run individually and separately, each in comparison to the N number of controls, rather than treating the multiple case samples as 'biological replicates' in a single analysis against the N controls (as would be performed by DE-kupl in the comparable workflow). Can the authors elaborate on this aspect and provide best recommendations? If such as multi-case sample situation is available, presumably it would enable for more direct comparisons to the DE-kupl method.

We have clarified in the text that each case is run individually versus N controls, even though the jobs are run in parallel. MINTIE does not currently support a multi-case analysis, and we do not have any biological or technical replicates available to test such a scenario. In such circumstances, we would recommend that users merge the fastq files from the replicates (assuming the sequencing characteristics are the same or highly similar), and run the sample as usual through the MINTIE pipeline.

Minor:

It would help for the authors to elaborate more on how they were able to infer the various TSV categories based on the results from these various programs. There was clearly a lot of work involved to do this, as most of these related software tools do not output lists of TSVs with categories assigned. While the methods do expand on how this was done, it would help to add a few sentences in the main text to emphasize that there is a lot of work here in downstream interpretation and classification into these different TSV event categories so it'll be better appreciated. Also, while I haven't had time to run MINTIE myself, it could be stated early on whether or not it provides such categorical lists of TSVs in its output or whether interpretation and analysis is required by the user.

Thank you for pointing this out. We have added a sentence alluding to the work involved in processing each tool’s individual outputs. To clarify, we do not specifically check the variant category in the simulation experiments, due to the very different output types from different programs (some of which omit categories all together). We instead looked for variants within the given gene or gene region, counting them as hits if they matched the simulation genes. To determine the accuracy of MINTIE’s classifications, we compared the simulation results to the truth data set and found that the variant type classifications were highly accurate (see Figure 3B).

As we compared a smaller number of callers on a smaller set of variants for the Leucegene validation, we were able to use more refined criteria for determining whether variant calls were accurate. These criteria included checking plausible classifications and requiring both ends of a fusion to be called. This is further detailed in the Methods.

p27 - adjust the wording to indicate that the Rapid RNA-seq pipeline is the St. Jude's pipeline and not something different.

We have clarified that this refers to St. Jude’s pipeline.

p11, line 29: ECCs used instead of ECs as elsewhere

ECs and ECCs are subtly different, as the former refers to the concept and the latter the counts belonging to each EC. To avoid confusion we have opted to remove the ECC acronym and retain only EC. This is now listed in the abbreviations section.

Reviewer #2: The manuscript entitled "MINTIE: identifying novel structural and splice variants in transcriptomes using RNA-seq data" describes an analytical pipeline which detect structural and splice variants in transcriptomes by a de novo assembly using SOAPdenovo-Trans of a case sample, merging assembled transcripts with the references assembled by CHESS v2.2, quantifying expression for both case and control samples using Salmon, and retain non-reference (termed "novel" by the authors) transcripts that are over-expressed only in case, and finally mapping the candidate transcripts to the genome and performing annotation. The accuracy of MINTIE was based on an analysis of simulated data which shows MINTIE can predict diverse classes of non-reference transcripts with accuracy comparable to those designed specifically for gene fusion or alternative splicing. Additionally, the authors presented MINTIE's recall rate of known fusions/FLT3 ITD in 77 AMLs and showcased novel

fusions in 87 pediatric B-ALLs as well as DMD fusion found in a rare disease sample. Overall, incorporating "control" samples which enables assessing differential expression in case sample versus "control" is the most unique design of MINTIE. However, the impact of this method, i.e. is uncertain. Specific comments are listed below.

Major issues:

a) The most desirable feature of MINTIE is its ability to capture aberrant splicing and gene fusions in a single analysis instead of running methods specifically designed for gene fusion and alternative splicing.

The superiority of MINTIE is demonstrated via its performance on simulated data of 15 different classes of variants presented in Figure 3. However, this result only shows that MANTIE has great performance on this simulated data and the design of the simulation, which gives high-coverage (50X coverage in a heterozygous fashion, total coverage of 100X, line 28-34, page 7) of the variant, do not match the expression profile of many pathogenic fusions or transcripts as these variants may not reach the 50X threshold in simulated RNA-seq data. Therefore, the statistics presented in Figure 3 may not reflect its performance on real cancer specimen. In fact, the false negative rate is somewhat reflected upon the less-than optimal performance of the 77 AMLs presented in the

section "MINTIE identifies known fusions and ITDs in Leucegene samples" as the missed CBFB-MYH11(2 out of 26) and KMT2A-PTD (8 out 24) were due to the lower expression level based on the authors evaluation. Comparison of MINTIE with other fusion/isoform detection methods on real disease samples is needed to convince the user that using MINTIE alone can achieve the same sensitivity as running a combo of fusion + isoform detection.

As mentioned in the response to reviewer 1’s second point, we have clarified that MINTIE’s primary strength is in detecting a broad array of variants that other variant callers miss and have removed the comment in the Discussion: “we propose that MINTIE could be used as a single tool to detect any large alterations, replacing the need to run multiple tools”. We do not claim that MINTIE can achieve the same sensitivity in variant finding as a combination of the most sensitive fusion and isoform detectors available. The extra benchmarking using competing tools on the Leucegene validation cohort shows this (see response to reviewer 1’s third point). We note that only three types of well known variants are tested with this data and MINTIE’s strength is in detecting novel events beyond fusions.

Moreover, MINTIE is able to discover rare variants that are more likely to be pathogenic by comparing to a set of controls, and provides interpretation of the variants by classifying them into types. This overcomes a major obstacle in interpretation and filtering of novel findings and differs, for example, from available methods that simply assemble alternative splicing.

We have addressed the question of the effect of varying coverage on MINTIE’s simulation results in reviewer 1’s second major point, which demonstrates that MINTIE is sensitive down to 5x variant coverage.

b) The authors need to present MINTIE's performance on solid and brain tumors, many of which have much lower tumor purity than leukemia samples presented in the manuscript. This will allow a more comprehensive analysis regarding MINTIE's performance on tumor samples.

To demonstrate MINTIE’s performance on solid tumours, we obtained the TCGA samples that had the novel RB1 variant we observed in our B-ALL samples. MINTIE found both RB1 variants in the BRCA and ESCA samples. The consensus purity estimate of the BRCA sample was 0.712 (Aran et al. Nature 2015); we could not find a corresponding purity estimate for the ESCA sample. MINTIE’s ability to detect both RB1 unpartnered fusions indicates that the approach is able to detect variants of interest in solid tumours. This is now described in the manuscript in the section “MINTIE identifies novel transcriptomic variants in B-ALL”.

MINTIE is able to work with samples of varying tumour purity. We note that the simulations countain heterozygous variant expression; i.e. each variant transcript has a corresponding unmodified transcript expressed at the same expression level. Additionally, variant transcripts that have a lower expression level than their wild type transcript, are readily detected. MINTIE calculates a variant allele frequency (VAF) metric based on this ratio, but does not threshold on it. We could detect the KMT2A-PTD down to a VAF of 0.058, suggesting that approximately 94% of the KMT2A expression in this sample does not contain the variant. We now make a note of this in the manuscript. The primary limitation on variant detection is adequate expression, not the proportion of expression which is affected by low tumour purity. Our simulation coverage experiments show robust variant detection by MINTIE down to 5x coverage (see reviewer 1’s major point 2).

In addition to the application of the method to leukaemia, as well as the breast and esophageal TCGA samples, we note that MINTIE has been successfully demonstrated on transcriptome sequencing of muscle biopsies from rare disease, suggesting it is likely to work across a broad range of tissues and disease.

c) SOAPdenovo assembly, or any other de novo assembly algorithms, may encounter issues when the fusion breakpoint occurs at the highly repetitive regions. For example, our test run of MANTIE using the Nalm6 cell line RNA-seq data (accessible as a demo sample for CICERO	at

[https://smex-ctp.trendmicro.com:443/wis/clicktime/v1/query?url=https%3a%2f%2fwww.stjuderesearch.org%2fsite%2flab%2fzhang%2fcicero&umid=41bae4ba-80a4-4fbd-a561-1bb541f3ea33&](https://smex-ctp.trendmicro.com/wis/clicktime/v1/query?url=https%3a%2f%2fwww.stjuderesearch.org%2fsite%2flab%2fzhang%2fcicero&umid=41bae4ba-80a4-4fbd-a561-1bb541f3ea33&auth=6ee66d642a212b82964c9073f0dd934b55317413-e9c8dc4c2cd0697d7ec7982fd44d15ee38869cbb) [auth=6ee66d642a212b82964c9073f0dd934b55317413-e9c8dc4c2cd0697d7ec7982fd44d15ee3](https://smex-ctp.trendmicro.com/wis/clicktime/v1/query?url=https%3a%2f%2fwww.stjuderesearch.org%2fsite%2flab%2fzhang%2fcicero&umid=41bae4ba-80a4-4fbd-a561-1bb541f3ea33&auth=6ee66d642a212b82964c9073f0dd934b55317413-e9c8dc4c2cd0697d7ec7982fd44d15ee38869cbb) [8869cbb](https://smex-ctp.trendmicro.com/wis/clicktime/v1/query?url=https%3a%2f%2fwww.stjuderesearch.org%2fsite%2flab%2fzhang%2fcicero&umid=41bae4ba-80a4-4fbd-a561-1bb541f3ea33&auth=6ee66d642a212b82964c9073f0dd934b55317413-e9c8dc4c2cd0697d7ec7982fd44d15ee38869cbb)) failed to detect the IGH-DUX4 fusion. In this test, we used 13 control RNA-seq (normal samples: 5 granulocytes, 5 monocytes, 3 total white blood cells) documented in MANTIE which produced a total of 1,870 variants. Limitation of the assembly algorithm needs to be noted so that the users are aware about the potential false negatives.

We have discussed this in the text:

*“MINTIE utilises de novo transcriptome assembly to reconstruct transcript sequences. While this has the advantage of allowing complex variants to be detected, ... the shortcomings of this approach also affect the method, such as difficulty with repetitive regions.”*

d) In the section "MINTIE detects a low number of background variants", the authors presented the variants detected a set of 23 non-cancer samples (Figure 4). However, there is a 10-fold differences within the samples and it is unclear what are the factors that contribute to the false positive rate.

To address this, and reviewer 1’s fourth point, we have further examined the influence of controls on reported variants in Figure 4 (now Figure 4B). We address this in Figure 4C in the “MINTIE detects a low number of background variants” section of the manuscript. The number of background variants will be influenced primarily by two factors: the number of controls used and the similarity of the case sample’s transcriptional profile relative to the control(s). Figure 4C shows how, on average, more controls results in fewer background variants. Figure 4B on the other hand, shows tests where, within each cell type, the total number of controls is constant, and thus differences in transcriptional profiles between the case and controls is likely the key factor leading to variability. Returning to Figure 4C, we can see that sample SRX372052 has high variability in the number of variant genes called when using different combinations of controls (but with a constant number of controls). By consulting a PCA plot (not shown), it was evident that when controls were selected that were closer to the case sample’s transcriptional profile, fewer background variants were detected, indicating that transcriptional profile differences between cases and controls are the most likely explanation for differences in variant number.

It should also be noted that nailing down the candidate fusions in the B-ALL samples presented in the section of "MINTIE identifies novel transcriptomic variants in B-ALL" has a dependency on knowledge-based ancillary data. First, control samples were from the same batch of the RNA-seq with a prior knowledge of RNA fusion status; second the candidate variants require filtering of candidate genes identified from prior pan-cancer studies. These constraints will greatly limit the applicability of MINTIE.

The candidate gene list was used primarily as a tool for prioritising variants for curation, which is standard practice. It is important to note that MINTIE runs transcriptome-wide, and filtering by a gene list is just one way variants can be prioritised (MINTIE also provides many other potential filters and prioritisation fields, such as variant type, significance, logFC and VAF).

Regarding prior knowledge in the case of the B-ALL samples, the fusion statuses were obtained primarily from cytogenetic tests rather than fusion detection solely from RNA-seq. Moreover, a possible use case for MINTIE is running it after fusion calling from the RNA-seq to find rare variants missed by fusion finders. It is thus reasonable that we may have prior knowledge of driver fusions. However, we acknowledge that a limitation of MINTIE is that variants commonly found within a cohort are likely to be missed unless normals are used as the control set. This is a design choice to be aware of, and we have noted this in the discussion section. It is also worth noting that one sample containing the RB1 variant was also present in the control cohort, indicating that the method is able to handle at least one true positive in the controls.

e) Novelty of the B-ALL findings presented in XXX is over-stated The unpartnered RB1 recurrent fusion was detected in the pan-TARGET study (Ma et al, Nature 2018) as loss-of-function structural variations (either by translocation or deletion) of RB1 in both T-lineage and B-lineage ALL. The variant can be easily explored on the recently published GenomePaint portal (<https://genomepaint.stjude.cloud/>, Zhou et al, Cancer Cell 2021). Similarly PAX5 PTD was first reported in 2007 (Mullighan et al, Nature 2007, Figure 2), and intra-genic deletion in ETV6 causing alternative splicing reported in the manuscript can be found in 2011 (Zhang et al, Blood 2011, Figure 4). Therefore, these findings are not novel.

We thank the reviewer for drawing our attention to these studies and have now cited them in the text of the manuscript and have revised the wording to indicate that these variants are not novel per se. These variants being seen previously strengthens the case for them being likely drivers detected by MINTIE. Regardless of their novelty, we emphasise that these are unusual variants that have been detected in an automated fashion using MINTIE.

It is worth noting that all the studies described identified these variants through DNA-based assays, while MINTIE has identified them through RNA-seq. Particularly, regarding the RB1 variant, MINTIE was able to find a fusion transcript, which was not previously reported, as the pan-TARGET study identified focal deletions in the gene, but did not characterise the effect on transcription of the gene. Finding a transcript correlated with the DNA event informs us that the transcript is stable, as well as informing the transcript structure (splice junctions and novel exons)--neither of which are easily inferred from DNA-based assays.

Minor issues

a) Figure 5. The y-axis label is difficult to read. The number of supporting reads for each splice junction is difficult to read. Panel B has two arcs representing the same splice junction but with varying read count. It will be good to document why there are different read counts in Figure legend

We have increased the text size of the figure to be more readable. The two arcs in panel B are actually slightly different splice junctions due to alternative splicing. This has been clarified in the figure caption.

b) References on the second paragraph of Results (page 5-6, starting with line 46) appear to be incorrect. For example: SOAPdenovo-Trans was first referenced on line 48, page 5. However, the reference (#28) it cited was referring to a different program DE-kupl. DE-kupl was cited using the old bioarchive paper instead of the Genome Biology publication. The references for CHESS and Salmon appear to be incorrect as well.

Thank you for pointing this out. This was caused by some stale references in our document and has now been fixed.

c) In the paragraph on page 14, the authors noted "The top recurrent genes were ETV6 (26 variants across 8 samples), IKZF2 (11 variants across 2 samples) and PAX5 (9 variants across 6 samples) (Supplementary Figure 5). It should be noted that the number of variants per sample is >1 in all three genes and in particular there are a total of 11 variants in 2 samples. Theauthors need to explain the genetic cause of these variants. In particular, IKZF2 was previously noted to have intra-genic deletion in hypodiploid ALL (Holmfeldt et al, Nature Genetics 2013). The high number of SVs presented in Supplementary Figure 5 is a surprise and the authors need to explain the potential genetic events leading to the high number of SVs in this gene in the two samples.

The results regarding IKZF2 have changed slightly due to updates to the MINTIE pipeline: we now observe 13 variants across three samples, where two of these samples contain 12 of the variants. We manually inspected these two samples. One sample contained three reported fusion events and a novel exon. These fusions all shared a single fusion break-end, with the matching break-ends mapping to three different intergenic loci within a 50kb region. As we observed with the DMD variant in rare disease, alternative isoforms may result from a single rearrangement. The second sample has several different splice variants called within IKZF2, some of which share break-ends. Similarly, this indicates alternative transcription. We also note that MINTIE can report multiple variants per event (see Figure 3B); hence unpartnered fusions are often reported as two variants: a fusion and a novel exon.

d) Memory requirement may exceed 90-100GB based on our test using the RNA-seq data from CICERO benchmark data set which requires 151-283 GB memory .We also noted false negative on MINTE in detecting lowly expressed gene fusions/ITD.

In our tests, we have observed in some cases maximum memory used can exceed 190GB due to the assembly step. We have added this point to the manuscript. We have also noted in the section “MINTIE identifies known fusions and ITDs in Leucegene samples”, that MINTIE may miss lowly expressed variants due to the requirements of de novo transcriptome assembly (6 of 24 variant contigs in the KMT2A cohort failed to be assembled).

**Second round of review**

**Reviewer 1**

The authors did a fantastic job at both responding to reviewers and revising the manuscript accordingly.

My only remaining comment is that the text "de novo assembled using SOAPdenovo-Trans48 v1.03 with kmers of 29, 49 and 69 (by default; in all samples presented in the paper, we used kmer sizes 49 and 79 unless otherwise indicated)." is confusing.  Why state the three kmer sizes and then indicate removing one (-29)  and changing another (69 -> 79)?  Perhaps just rewording this for clarity is all that is needed.

**Reviewer 2**

The authors have addressed all the questions raised in the previous review.
